# Supplementary material for: Biomimetic versus arbitrary motor control strategies for bionic hand skill learning
Source: Nat Hum Behav. 2024 Mar 18;8(6):1108–23. doi: 10.1038/s41562-023-01811-6 (PMC11199138; doi:10.1038/s41562-023-01811-6)
Supplement: Supplementary file 1 — Supplementary Figs. 1–3 and Tables 1 and 2. [file 41562_2023_1811_MOESM1_ESM.pdf]

nature human behaviour

Article

https://doi.org/10.1038/s41562-023-01811-6

# Biomimetic versus arbitrary motor control strategies for bionic hand skill learning

In the format provided by the authors and unedited

## Supplementary Figures and Tables

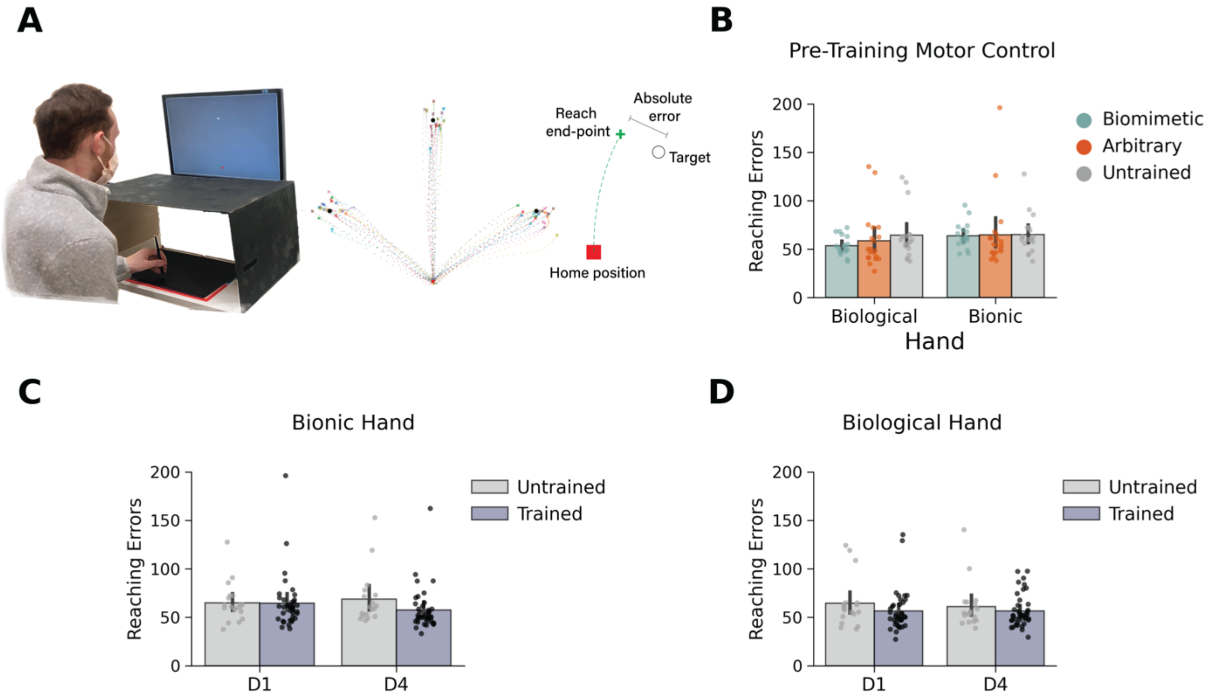


**Supplementary Figure 1. Similar motor abilities between groups when using biological and bionic hands. (A)** A participant performing ballistic reaches (with no corrective movements) to virtual targets using a digitizing tablet and stylus. The task was performed with either participant's left biological hand or the bionic hand locked around the stylus (see Methods for a description). Not shown in the figure image, during the task, all participants wore a barber cape over the apparatus to remove any potential visual feedback of their arm/hand. Participants performed 60 reaches with each hand (biological and bionic) to 3 different virtual targets (example of a participant reaches shown in panel A). The primary measure of motor ability we quantified is the average absolute error between reach end points and the virtual target location. **(B)** Before training, all groups (n=57) made similar reaching errors when reaching with their biological hand or the bionic hand. **(C)** After-training, trained participants (biomimetic and arbitrary combined; n=40) made smaller reaching errors, on average, than untrained participants (n=18) when using the bionic hand (two-tailed Mann Whitney: $W$=498.0, $p$=0.020, $r_{rb}$=0.38, 95% CI [0.08 0.62]), but similar errors before training (two-tailed Mann Whitney: $W$=397.0, $p$=0.551, $r_{rb}$=0.10, 95% CI [-0.21 0.39], $BF_{10}$=0.29). **(D)** When using their biological hand, trained (n=38) and untrained (n=19) participants showed similar reaching errors both before (two-tailed Mann Whitney: $W$=444.0, $p$=0.164, $r_{rb}$=0.23, 95% CI [-0.08 0.50], $BF_{10}$=0.45) and after training (two-tailed Mann Whitney: $W$=409.0, $p$=0.418, $r_{rb}$=0.13, 95% CI [-0.18 0.43], $BF_{10}$=0.44). For panels B-D, circles depict individual subject means (across relevant items) and values indicate group means ± standard error.

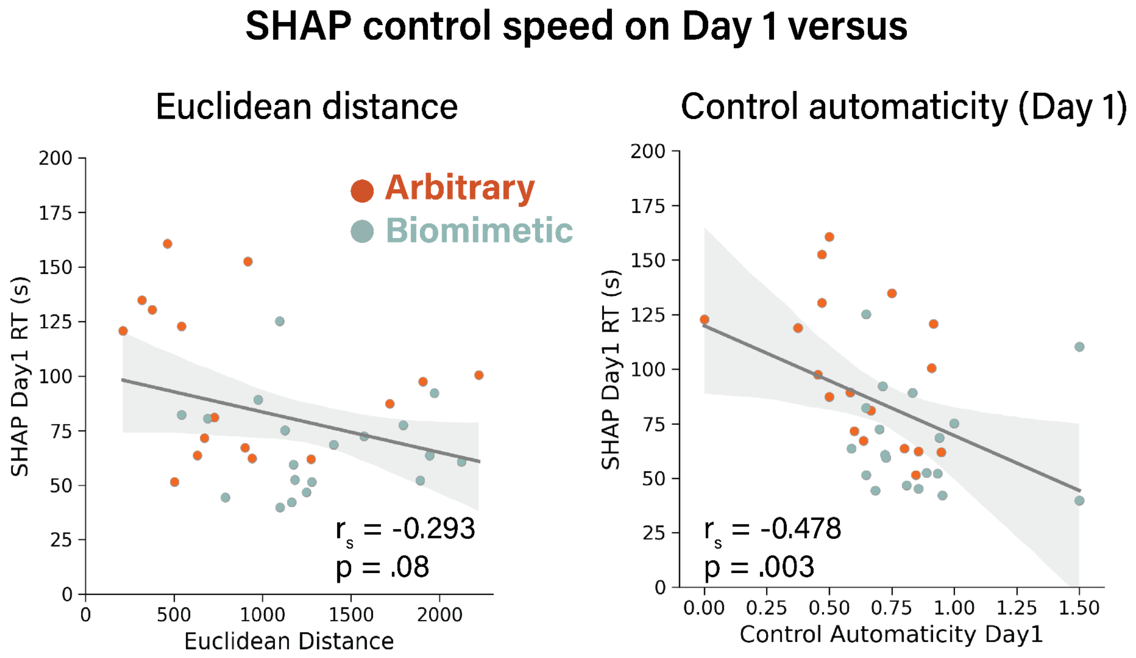


**Supplementary Figure 2. Relationship between Day1 measures.** (**Left**) There was not a significant relationship between all trained participants (n=35) SHAP Day 1 control speed and the euclidean distance between the first two trained gestures [used on the task; $r_s$=-0.293, p=0.088 (two-tailed)]. (**Right**) There was a significant relationship between all trained participants (n=38) Day 1 control automaticity and SHAP Day 1 control speed [$r_s$=-0.478, p=0.003 (two-tailed)], such that more automatic control reflected faster performance on the SHAP. The shaded grey area reflects the 95% confidence interval of the regression line.

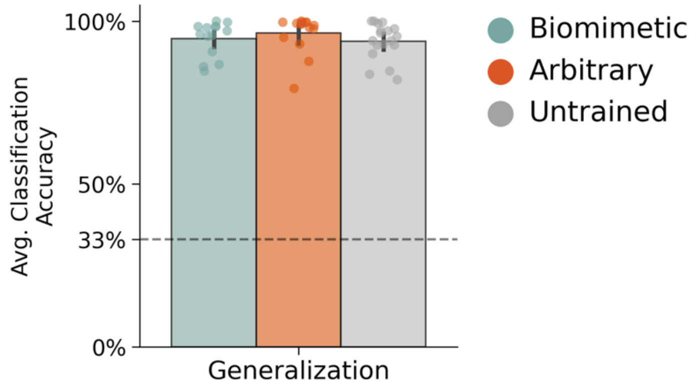


**Supplementary Figure 3. Similar classification accuracy for all groups during the generalization session.** Average classification accuracy for the generalization control mappings was calculated in the same way as Figure 5. Circles depict individual subject means and values indicate group means ± standard error.

**Supplementary Video 1. Video examples of the tasks.**

| **Body Ownership** |
|---|
| 1. “It seems like the robotic hand belongs to me” |
| 2. “It seems like the robotic hand is my hand” |
| 3. “It seems like the robotic hand is part of my body” |
| 4. “It feels like my robotic hand is a foreign body” |
| 5. “It feels like my robotic hand is fused with my body” |
| 6. “It seems like I have three hands” |
| **Agency** |
| 1. “It seems like I can move the robotic hand fingers if I want” |
| 2. “It seems like I am in control of the robotic hand” |
| **Body image** |
| 1. “It seems like I am looking directly at my own hand, rather than a robotic hand” |

**Supplementary Table 1. Embodiment questionnaire statements divided into three categories.**

| **Days** | **Condition - Measure** | **Statistical Test** | **Result** |
|---|---|---|---|
| **Speed – Southampton Hand Assessment Procedure** | | | |
| D1,D2,D3,D4 | Object1 – Completion Time | RmANOVA:<br>Days: 4<br>Groups: 2 | Day x Group:<br>$F_{(3,114)}$=6.892, p<0.001<br>Day:<br>$F_{(3,114)}$=67.970, p<0.001<br>Group:<br>$F_{(1,38)}$=14.031, p<0.001 |
| | | Mann Whitney | D1: W=85.0, p=0.001<br>D2: W=67.0, p<0.001<br>D3: W=93.50, p=0.004<br>D4: W=96.0, p=0.004 |
| | | Bayes Mann-Whitney | D1: $BF_{10}$=15.961<br>D2: $BF_{10}$=20.952<br>D3: $BF_{10}$=8.145<br>D4: $BF_{10}$=4.938 |
| Generalization | Object1 – Completion Time | ANOVA:<br>Groups: 3 | $F_{(2,54)}$ = 1.212, p = = .306 |
| | | Mann Whitney | Ctrl to Bio: W=202.0, p=0.534<br>Ctrl to Arb: W=239.0, p=0.039<br>Bio to Arb: W=250.0, p=0.095 |
| | | Bayes Mann-Whitney | Ctrl to Bio: $BF_{10}$ = .383<br>Ctrl to Arb: $BF_{10}$ = 1.505<br>Bio to Arb: $BF_{10}$ = .873 |
| D2,D3,D4 | Object2 – Completion Time | RmANOVA:<br>Days: 3<br>Groups: 2 | Day x Group:<br>$F_{(2,76)}$ = 3.491, p=0.035<br>Day:<br>$F_{(2,76)}$ = 47.583, p < .001<br>Group:<br>$F_{(1,38)}$ = 1.087, p = .304 |
| | | Mann Whitney | D2: W=263.0, p=0.088<br>D3: W=232.0, p=0.390<br>D4: W=175.0, p=0.524 |
| | | Bayes Mann-Whitney | D2: $BF_{10}$=0.722<br>D3: $BF_{10}$=0.384<br>D4: $BF_{10}$=0.453 |
| D3,D4 | Object3 – Completion Time | RmANOVA:<br>Days: 2<br>Groups: 2 | Day x Group:<br>$F_{(1,38)}$=0.375, p=0.544<br>Day: |

| | | | |
|---|---|---|---|
| | | | $F_{(1,38)}$=5.196, p=0.028<br>Group:<br>$F_{(1,38)}$=0.185, p=0.670 |
| | | Mann Whitney | D3: W=177.0, p=0.555<br>D4: W=158.0, p=0.270 |
| | | Bayes Mann-Whitney | D3: $BF_{10}$=0.362<br>D4: $BF_{10}$=0.472 |
| **Dexterity - Virtual eggs test** | | | |
| D1,D2,D3,D4 | Number of successful (unbroken) eggs transferred | RmANOVA:<br>Days: 4<br>Groups:2 | Day X Group:<br>$F_{(3,108)}$ = 1.157, p = .330<br>Day:<br>$F_{(3,108)}$ = 12.960, p < .001<br>Group:<br>$F_{(1,36)}$ = .363, p = .550 |
| | | Mann Whitney | D1: W=185.50, p=0.897<br>D2: W=220.00, p=0.209<br>D3: W=216.0, p=0.468<br>D4: W=209.0, p=0.598 |
| | | Bayes Mann-Whitney | D1: $BF_{10}$=0.453<br>D2: $BF_{10}$=0.715<br>D3: $BF_{10}$=0.413<br>D4: $BF_{10}$=0.319 |
| Generalization | Number of successful (unbroken) eggs transferred | ANOVA:<br>Groups: 3 | $F_{(2,54)}$ = 7.787, p =0.001 |
| | | Bayesian ANOVA | $BF_{10}$=32.417 |
| | | Mann Whitney | Ctrl to Arb: W=88.50, p=0.010<br>Ctrl to Bio: W=170.0, p=0.716<br>Bio to Arb: W=104.50, p=0.013 |
| | | Bayes Mann Whitney | Ctrl to Bio: $BF_{10}$=0.351<br>Ctrl to Arb: $BF_{10}$=2.530<br>Bio to Arb: $BF_{10}$=2.521 |
| D1,D2,D3,D4 | Percentage of total egg transfers that were successful (unbroken) | RmANOVA:<br>Days: 4<br>Groups:2 | Day X Group:<br>$F_{(3,105)}$ = 0.896, p = 0.446<br>Day:<br>$F_{(3,105)}$ = 8.965, p < 0.001<br>Group:<br>$F_{(1,35)}$ = .005, p = 0.945 |
| | | Mann Whitney | D1: W=221.50, p=0.740<br>D2: W=253.50, p=0.143<br>D3: W=200.0, p=0.789<br>D4: W=218.0, p=0.631 |
| | | Bayes Mann-Whitney | D1: $BF_{10}$=0.322<br>D2: $BF_{10}$=0.615<br>D3: $BF_{10}$=0.315<br>D4: $BF_{10}$=0.313 |
| D1,D2,D3,D4 | Applied pressure | RmANOVA:<br>Days: 4<br>Groups:2 | Day X Group:<br>$F_{(3,102)}$ = 0.277, p=0.842<br>Day:<br>$F_{(3,102)}$ = 5.476, p=0.002<br>Group:<br>$F_{(1,34)}$ = .090, p=0.765 |
| | | Mann Whitney | D1: W=205.0, p=0.904<br>D2: W=200.0, p=0.792<br>D3: W=173.0, p=0.851<br>D4: W=212.0, p=0.550 |
| | | Bayes Mann-Whitney | D1: $BF_{10}$=0.335<br>D2: $BF_{10}$=0.34<br>D3: $BF_{10}$=0.33<br>D4: $BF_{10}$=0.35 |
| **Gesture Switching – Block stacking** | | | |
| D2,D3,D4 | 2 gesture version – completion time | RmANOVA:<br>Days: 3<br>Groups: 2 | Day X Group:<br>$F_{(2,76)}$=3.602, *p*=0.032<br>Day:<br>$F_{(2,76)}$ =13.766, *p*<0.001<br>Group:<br>$F_{(1,38)}$=0.044, *p*=0.835 |
| | | Mann Whitney | D2: W=223.0, *p*=0.537<br>D3: W=194.0, *p*=0.689<br>D4: W=154.0, *p*=0.149 |
| | | Bayes Mann Whitney | D2: $BF_{10}$=0.379<br>D3: $BF_{10}$=0.319 |

| | | | |
|---|---|---|---|
| | | | D4: $BF_{10}$=0.844 |
| D3 | 3 gesture version – completion time | Mann Whitney<br>Bayes Mann-Whitney | W=172.0, $p$=0.646<br>$BF_{10}$=0.349 |
| **Control Automaticity - Cognitive Load** | | | |
| D1,D4 | Control Automaticity (blocks stacked with load divided by blocks stacked without load) | RmANOVA:<br>Days: 2<br>Groups: 2<br><br>Mann Whitney<br><br>Bayes Mann-Whitney | Day X Group:<br>$F_{(1,38)}$=9.896, $p$=0.003<br>Day:<br>$F_{(1,38)}$=5.475, $p$=0.022<br>Group:<br>$F_{(1,38)}$=1.753, $p$=0.193<br><br>D1: W=286.50, $p$=0.019<br>D4: W=176.0, $p$=0.533<br><br>D1: $BF_{10}$=286.50<br>D4: $BF_{10}$=0.365 |
| **Control Automaticity - Subjective Control Difficulty** | | | |
| D1, D2, D3, D4 | Raw scores | RmANOVA:<br>Days: 4<br>Groups: 2<br><br>Mann Whitney<br><br>Bayes Mann-Whitney | Day X Group:<br>$F_{(3,114)}$=2.924, p=0.037<br>Day:<br>$F_{(3,114)}$=21.298, p<0.001<br>Group:<br>$F_{(1,38)}$=0.041, p=0.840<br><br>D1: W=156.0, $p$=0.159<br>D2: W=242.50, $p$=0.397<br>D3: W=197.50, $p$=0.956<br>D4: W=232.0, $p$=0.566<br><br>D1: $BF_{10}$=0.824<br>D2: $BF_{10}$=0.382<br>D3: $BF_{10}$=0.312<br>D4: $BF_{10}$=0.377 |
| D4, Generalization | Raw scores | RmANOVA:<br>Days: 2<br>Groups: 2<br><br>Mann Whitney<br><br>Bayes Mann-Whitney<br><br>Wilcoxon signed-rank<br><br>Bayes Wilcoxon | Day X Group:<br>$F_{(1,38)}$=6.857, p=0.013<br>Day:<br>$F_{(1,38)}$=2.469, p=0.124<br>Group:<br>$F_{(1,38)}$=7.626, p=0.009<br><br>D4: W=232.0, $p$=0.566<br>Generalization: W=310.0, $p$=0.003<br><br>D4: $BF_{10}$=0.377<br>Generalization: $BF_{10}$=30.395<br><br>Bio: D4 vs. Gen: W=15.50, $p$=0.012<br>Arb: D4 vs. Gen: W=104.0, $p$=0.427<br><br>Bio: D4 vs. Gen: $BF_{10}$=9.539<br>Arb: D4 vs. Gen: $BF_{10}$=0.279 |
| Generalization | Raw scores | ANOVA:<br>Groups: 3<br><br>Mann Whitney<br><br>Bayes Mann-Whitney | $F_{(2,55)}$ = 9.178, p = < .001<br><br>Ctrl to Arb: W=287.50, $p$=0.002<br>Ctrl to Bio: W=194.0, $p$=0.688<br>Bio to Arb: W=310.0, $p$=0.003<br><br>Ctrl to Bio: $BF_{10}$=0.331<br>Ctrl to Arb: $BF_{10}$=28.721<br>Bio to Arb: $BF_{10}$=30.395 |
| **Sense of Embodiment** | | | |
| Pre, Post | Raw scores | RmANOVA:<br>Days: 2<br>Groups: 2 (trained vs. untrained)<br><br>Mann-Whitney<br><br>Bayes Mann-Whitney<br><br>Run for each category separately | <u>Body Ownership</u><br>Day X Group:<br>$F_{(1,58)}$=7.621, p=0.008<br>Day:<br>$F_{(1,58)}$=9.158, p=0.004<br>Group:<br>$F_{(1,58)}$=1.359, p=0.248<br><br>Pre: W=412.50, $p$=0.720; $BF_{10}$=0.291<br>Post: W=262.50, $p$=0.044; $BF_{10}$=1.861<br><br><u>Agency</u><br>Day X Group:<br>$F_{(1,58)}$=39.323, p<0.001<br>Day:<br>$F_{(1,58)}$=18.082, p<0.001<br>Group:<br>$F_{(1,58)}$=8.530, p=0.005<br><br>Pre: W=461.50, $p$=0.252; $BF_{10}$=0.581<br>Post: W=76.50, $p$<0.001; $BF_{10}$=124.245 |

| | | | |
|---|---|---|---|
| | | | Visual Appearance<br>Day X Group:<br>$F_{(1,58)}$=15.998, p<0.001<br>Day:<br>$F_{(1,58)}$=5.359, p=0.024<br>Group:<br>$F_{(1,58)}$=0.217, p=0.643<br><br>Pre: W=501.50, *p*=0.070; $BF_{10}$=0.978<br>Post: W=184.50, *p*<0.001; $BF_{10}$=34.825 |
| Pre and Post | Post-Pre difference score | Mann Whitney<br>Groups: 2 (trained vs. untrained)<br><br>Mann-Whitney<br>Groups: 2 (bio vs. arb)<br><br>Bayes Mann-Whitney<br>Groups: 2 (bio vs. arb) | Body ownership: W=263.0, *p*=0.045<br>Agency: W=81.0, *p<0.001*<br>Visual appearance: W=169.50, *p*<0.001<br><br>Body ownership: W=266.50, *p*=0.143<br>Agency: W=193.50, *p*=0.675<br>Visual appearance: W=228.50, *p*=0.632<br><br>Body Ownership: $BF_{10}$=0.532<br>Agency: $BF_{10}$=0.385 |
| **Classification Accuracy** | | | |
| D1 | 3-motion-class (avg. of rest, open, close) | Mann Whitney<br><br>Bayes Mann-Whitney | *W*=167.0, *p*=0.631<br>$BF_{10}$=0.375 |
| D1, D4 | 3-motion-class (avg. of rest, open, close) | RmANOVA:<br>Days: 2<br>Groups: 2 (bio vs. arb)<br><br>Mann-Whitney<br><br>Bayes Mann-Whitney | Day X Group:<br>$F_{(1,31)}$=2.622, p=0.116<br>Day:<br>$F_{(1,31)}$=5.647, p=0.024<br>Group:<br>$F_{(1,31)}$=0.001, p=0.970<br><br>Pre: W=167.0, *p*=0.631<br>Post: W=121.50, *p*=0.437<br><br>Pre: $BF_{10}$=0.375<br>Post: $BF_{10}$=0.371 |
| D4 | 5-motion-class (avg. of rest, open, close, pinch, tripod) | Mann-Whitney<br><br>Bayes Mann-Whitney | W=117.50, *p*=0.517<br><br>$BF_{10}$=0.422 |
| Generalization | 3-motion-class (avg. of rest, open, close) | ANOVA:<br>Groups: 3 | $F_{(2,44)}$=0.874, p=0.424 |
| **EMG gestural structure** | | | |
| D4 | Euclidean distance for all trained gestures | Mann Whitney<br><br>Bayes Mann-Whitney | *W*=131.0, *p*=0.346<br><br>$BF_{10}$=0.730 |
| D1 | Euclidean distance for first two trained gestures | Mann Whitney<br><br>Bayes Mann-Whitney | *W*=250.0, *p*=0.004<br><br>$BF_{10}$=4.028 |
| **Motor control** | | | |
| Pre | Bionic hand - Absolute error | ANOVA:<br>Groups: 3<br><br>Bayesian ANOVA:<br>Groups: 3 | $F_{(2,54)}$=0.009, p=0.991<br><br>$BF_{10}$=0.14 |
| Pre | Biological hand - Absolute error | ANOVA:<br>Groups: 3<br><br>Bayesian ANOVA:<br>Groups: 3 | *$F_{(2,54)}$*=1.012, *p*=0.370<br><br>$BF_{10}$=0.291 |
| Pre and Post | Bionic hand – Absolute error | RmANOVA:<br>Days: 2<br>Groups: 2 (trained vs. untrained)<br><br>Mann Whitney | Day X Group:<br>$F_{(1,50)}$=4.010, p=0.051<br>Day:<br>$F_{(1,50)}$=2.522, p=0.118<br>Group:<br>$F_{(1,50)}$ =1.725, p=0.195<br><br>Pre: W=397.0, p=0.551<br>Post: W=498.0, p=0.020 |

**Supplementary Table 2. All statistical analyses.**
