# Supplementary material for: Biomimetic versus arbitrary motor control strategies for bionic hand skill learning
Source: Nat Hum Behav. 2024 Mar 18;8(6):1108–23. doi: 10.1038/s41562-023-01811-6 (PMC11199138; doi:10.1038/s41562-023-01811-6)
Supplement: Supplementary file 2 — Reporting Summary [file 41562_2023_1811_MOESM2_ESM.pdf]

nature portfolio

Corresponding author(s): Hunter Schone

Last updated by author(s): Dec 13, 2023

Reporting Summary

Nature Portfolio wishes to improve the reproducibility of the work that we publish. This form provides structure for consistency and transparency in reporting. For further information on Nature Portfolio policies, see our Editorial Policies and the Editorial Policy Checklist.

Statistics

For all statistical analyses, confirm that the following items are present in the figure legend, table legend, main text, or Methods section.

n/a	Confirmed	
☐	☒	The exact sample size (n) for each experimental group/condition, given as a discrete number and unit of measurement
☐	☒	A statement on whether measurements were taken from distinct samples or whether the same sample was measured repeatedly
☐	☒	The statistical test(s) used AND whether they are one- or two-sided<br>*Only common tests should be described solely by name; describe more complex techniques in the Methods section.*
☐	☒	A description of all covariates tested
☐	☒	A description of any assumptions or corrections, such as tests of normality and adjustment for multiple comparisons
☐	☒	A full description of the statistical parameters including central tendency (e.g. means) or other basic estimates (e.g. regression coefficient) AND variation (e.g. standard deviation) or associated estimates of uncertainty (e.g. confidence intervals)
☐	☒	For null hypothesis testing, the test statistic (e.g. F, t, r) with confidence intervals, effect sizes, degrees of freedom and P value noted<br>*Give P values as exact values whenever suitable.*
☐	☒	For Bayesian analysis, information on the choice of priors and Markov chain Monte Carlo settings
☐	☒	For hierarchical and complex designs, identification of the appropriate level for tests and full reporting of outcomes
☐	☒	Estimates of effect sizes (e.g. Cohen's d, Pearson's r), indicating how they were calculated

Our web collection on statistics for biologists contains articles on many of the points above.

Software and code

Policy information about availability of computer code

Data collection	EMG data was recorded using the Coapt COMPLETE CONTROL Gen2 pattern recognition system [Coapt, LLC; firmware vl.27; software v.l.1.9]. Presentation software included PsychoPy (v2021.1.1).
Data analysis	All statistical analyses were performed using JASP (v0.14). All data was analyzed using custom Python (version 3) scripts. Code used in the study can be accessed at https://github.com/hunterschone/ProControl. All code will be made available prior to publication.

For manuscripts utilizing custom algorithms or software that are central to the research but not yet described in published literature, software must be made available to editors and reviewers. We strongly encourage code deposition in a community repository (e.g. GitHub). See the Nature Portfolio guidelines for submitting code & software for further information.

Data

Policy information about availability of data

All manuscripts must include a data availability statement. This statement should provide the following information, where applicable:

- Accession codes, unique identifiers, or web links for publicly available datasets
- A description of any restrictions on data availability
- For clinical datasets or third party data, please ensure that the statement adheres to our policy

Pre-registered study predictions and methods, as well as the data used in the study, can be accessed at: https://osf.io/ 3m592/.

Research involving human participants, their data, or biological material

Policy information about studies with human participants or human data. See also policy information about sex, gender (identity/presentation), and sexual orientation and race, ethnicity and racism.

Reporting on sex and gender	Sixty-one healthy volunteers (40 females; mean age= 24.8 ± 0.66; all right handed) were recruited from the National Institute of Health community and the Washington DC metro area and were randomly assigned to one of the following study groups: biomimetic (n = 21; 14 females; mean age 23.9 ± 0.57), arbitrary (n = 21; 12 females; mean age 25.9 ± 1.28) or untrained (n = 19; 14 females; mean age 24.6 ± 1.41). Information on sex was self-reported by the volunteers.
Reporting on race, ethnicity, or other socially relevant groupings	*Please specify the socially constructed or socially relevant categorization variable(s) used in your manuscript and explain why they were used. Please note that such variables should not be used as proxies for other socially constructed/relevant variables (for example, race or ethnicity should not be used as a proxy for socioeconomic status).*<br>*Provide clear definitions of the relevant terms used, how they were provided (by the participants/respondents, the researchers, or third parties), and the method(s) used to classify people into the different categories (e.g. self-report, census or administrative data, social media data, etc.)*<br>*Please provide details about how you controlled for confounding variables in your analyses.*
Population characteristics	Sixty-one healthy volunteers (40 females; mean age= 24.8 ± 0.66; all right handed) were recruited from the National Institute of Health community and the Washington DC metro area and were randomly assigned to one of the following study groups: biomimetic (n = 21; 14 females; mean age 23.9 ± 0.57), arbitrary (n = 21; 12 females; mean age 25.9 ± 1.28) or untrained (n = 19; 14 females; mean age 24.6 ± 1.41). Information on sex was self-reported by the volunteers. All participants had no known motor disorders, as determined by a nurse practitioner.
Recruitment	All participants were recruited from the National Institute of Health community and the Washington DC metro area via an NIH research participant online database and word of mouth.
Ethics oversight	The study and its experimental procedures were approved by the NIH Institutional Review Board (NCT00001360, 93M-0170).

Note that full information on the approval of the study protocol must also be provided in the manuscript.

Field-specific reporting

Please select the one below that is the best fit for your research. If you are not sure, read the appropriate sections before making your selection.

☐ Life sciences ☒ Behavioural & social sciences ☐ Ecological, evolutionary & environmental sciences

For a reference copy of the document with all sections, see nature.com/documents/nr-reporting-summary-flat.pdf

Behavioural & social sciences study design

All studies must disclose on these points even when the disclosure is negative.

Study description	Quantitative experimental
Research sample	Sixty-one healthy volunteers (40 females; mean age= 24.8 ± 0.66; all right handed) were recruited from the National Institute of Health community and the Washington DC metro area and were randomly assigned to one of the following study groups: biomimetic (n = 21; 14 females; mean age 23.9 ± 0.57), arbitrary (n = 21; 12 females; mean age 25.9 ± 1.28) or untrained (n = 19; 14 females; mean age 24.6 ± 1.41). The sample size is based on a power analysis for learning effects with an artificial body-part (Kieliba et al., 2021, Sci Robotic) and was specified in the studies pre-registration.
Sampling strategy	Our sample sizes were based on a power analysis for learning effects with an artificial body part, (see Kieliba et al., 2021, Sci. Robotics), as well as what was technically feasible to accommodate the most number of sessions for the most number of subjects.
Data collection	There are a variety of data-types included in the study. EMG data was recorded using the Coapt COMPLETE CONTROL Gen2 pattern recognition system (Coapt, LLC; firmware vl.27; software v.l.1.9]. Motor control data was collected using a digitizing tablet (42.6 by 28.4 cm, lntuos Pro Large; Wacom, Vancouver, WA). All bionic hand training sessions were filmed. Bionic hand task data (speed, dexterity, gesture switching, control automaticity) was recorded by pen and paper and validated offline by video analyses. For all sessions, a single researcher and the research participant were present. Because the testing required the researcher to know which control strategy each participant belonged to, the researcher could not be blind to which training group a participant belonged to.
Timing	All data collection took place between May 3, 2021 to May 2nd, 2022.
Data exclusions	One participant's speed data was excluded, due to a technical issue with their Coapt EMG controller.
Non-participation	Two additional participants were recruited, but not included in the present study due to dropping out prior to competing the study.
Randomization	Volunteers were randomly assigned to the study groups.

Reporting for specific materials, systems and methods

We require information from authors about some types of materials, experimental systems and methods used in many studies. Here, indicate whether each material, system or method listed is relevant to your study. If you are not sure if a list item applies to your research, read the appropriate section before selecting a response.

Materials & experimental systems

n/a	Involved in the study
☒	☐ Antibodies
☒	☐ Eukaryotic cell lines
☒	☐ Palaeontology and archaeology
☒	☐ Animals and other organisms
☒	☐ Clinical data
☒	☐ Dual use research of concern
☒	☐ Plants

Methods

n/a	Involved in the study
☒	☐ ChIP-seq
☒	☐ Flow cytometry
☒	☐ MRI-based neuroimaging

Plants

Seed stocks

Report on the source of all seed stocks or other plant material used. If applicable, state the seed stock centre and catalogue number. If plant specimens were collected from the field, describe the collection location, date and sampling procedures.

Novel plant genotypes

Describe the methods by which all novel plant genotypes were produced. This includes those generated by transgenic approaches, gene editing, chemical/radiation-based mutagenesis and hybridization. For transgenic lines, describe the transformation method, the number of independent lines analyzed and the generation upon which experiments were performed. For gene-edited lines, describe the editor used, the endogenous sequence targeted for editing, the targeting guide RNA sequence (if applicable) and how the editor was applied.

Authentication

Describe any authentication procedures for each seed stock used or novel genotype generated. Describe any experiments used to assess the effect of a mutation and, where applicable, how potential secondary effects (e.g. second site T-DNA insertions, mosiacism, off-target gene editing) were examined.
